# Dose-response relationships using brain–computer interface technology impact stroke rehabilitation

**DOI:** 10.3389/fnhum.2015.00361

**Published:** 2015-06-23

**Authors:** Brittany M. Young, Zack Nigogosyan, Léo M. Walton, Alexander Remsik, Jie Song, Veena A. Nair, Mitchell E. Tyler, Dorothy F. Edwards, Kristin Caldera, Justin A. Sattin, Justin C. Williams, Vivek Prabhakaran

**Affiliations:** ^1^Department of Radiology, University of Wisconsin Hospital & Clinics, University of Wisconsin-Madison, MadisonWI, USA; ^2^Medical Scientist Training Program, University of Wisconsin-Madison, MadisonWI, USA; ^3^Neuroscience Training Program, University of Wisconsin-Madison, MadisonWI, USA; ^4^Department of Biomedical Engineering, University of Wisconsin-Madison, MadisonWI, USA; ^5^Department of Kinesiology and Department of Medicine, University of Wisconsin-Madison, MadisonWI, USA; ^6^Department of Neurology, University of Wisconsin-Madison, MadisonWI, USA; ^7^Department of Orthopedics and Rehabilitation, University of Wisconsin-Madison, MadisonWI, USA; ^8^Department of Neurosurgery, University of Wisconsin-Madison, MadisonWI, USA; ^9^Department of Psychology and Department of Psychiatry, University of Wisconsin-Madison, MadisonWI, USA

**Keywords:** dose-response, brain–computer interface, stroke rehabilitation, BCI therapy, UE motor recovery, fMRI

## Abstract

Brain–computer interfaces (BCIs) are an emerging novel technology for stroke rehabilitation. Little is known about how dose-response relationships for BCI therapies affect brain and behavior changes. We report preliminary results on stroke patients (*n* = 16, 11 M) with persistent upper extremity motor impairment who received therapy using a BCI system with functional electrical stimulation of the hand and tongue stimulation. We collected MRI scans and behavioral data using the Action Research Arm Test (ARAT), 9-Hole Peg Test (9-HPT), and Stroke Impact Scale (SIS) before, during, and after the therapy period. Using anatomical and functional MRI, we computed Laterality Index (LI) for brain activity in the motor network during impaired hand finger tapping. Changes from baseline LI and behavioral scores were assessed for relationships with dose, intensity, and frequency of BCI therapy. We found that gains in SIS Strength were directly responsive to BCI therapy: therapy dose and intensity correlated positively with increased SIS Strength (*p* ≤ 0.05), although no direct relationships were identified with ARAT or 9-HPT scores. We found behavioral measures that were not directly sensitive to differences in BCI therapy administration but were associated with concurrent brain changes correlated with BCI therapy administration parameters: therapy dose and intensity showed significant (*p* ≤ 0.05) or trending (0.05 < *p* < 0.1) negative correlations with LI changes, while therapy frequency did not affect LI. Reductions in LI were then correlated (*p* ≤ 0.05) with increased SIS Activities of Daily Living scores and improved 9-HPT performance. Therefore, some behavioral changes may be reflected by brain changes sensitive to differences in BCI therapy administration, while others such as SIS Strength may be directly responsive to BCI therapy administration. Data preliminarily suggest that when using BCI in stroke rehabilitation, therapy frequency may be less important than dose and intensity.

## Introduction

There is a growing need for the study and development of advancements in the field of stroke rehabilitation. Each year nearly 800,000 individuals in the United States suffer from a new stroke (Go et al., 2014), and even with standard rehabilitative approaches up to half of stroke survivors continue to live with some neurological impairment (Kelly-Hayes et al., 2003). Reductions in stroke mortality (Lackland et al., 2014) and continued growth of the aging population have contributed to an increasing number of stroke survivors for whom new options in rehabilitation are needed to facilitate further recovery of function, independence, and improvements in overall quality of life.

One emerging approach to stroke rehabilitation uses brain–computer interface (BCI) technology. These devices allow for real-time feedback of neural activity, which can then be used to train and/or modulate neural activity while performing guided rehabilitative tasks. Early studies of BCI devices being used for rehabilitation have suggested the potential for meaningful gains in motor function to be achieved even after traditional therapies have failed to facilitate full recovery (Broetz et al., 2010; Prasad et al., 2010; Caria et al., 2011; Shindo et al., 2011; Liu et al., 2012; Takahashi et al., 2012; Ang et al., 2014a,b; Mukaino et al., 2014; Ono et al., 2014; Young et al., 2014a). The development of BCI devices to address persistent motor impairment after stroke may hold promise for additional meaningful recovery in stroke survivors (Young et al., 2014d). Deficits in motor function are a frequent source of persistent impairment after stroke. In particular, applications intended to facilitate improvements in upper extremity motor function are an area of great need, as the upper extremity is more severely involved than the lower extremity in most stroke survivors with motor impairments (Shelton and Reding, 2001).

Studies of traditional standard therapies for stroke rehabilitation have shown increased therapy time and repetitions during therapy to be associated with improvements in outcomes (Nugent et al., 1994; Langhorne et al., 1996; Cifu and Stewart, 1999; Kwakkel et al., 1999; van der Lee et al., 2001b; Haines et al., 2011). Some studies of emerging therapies such as robot-assisted therapy have suggested similar relationships in which increased therapy dose or intensity is associated with improved outcomes (Burgar et al., 2011; Hsieh et al., 2012). However, other studies of emerging therapies such as neuromuscular electrical stimulation or constraint-induced movement therapy (CIMT) have found that increased therapy time is not always superior (Hsu et al., 2010), and in some cases higher intensity therapy has produced less improvement than lower intensity therapy administration (Dromerick et al., 2009). Thus, it is unclear whether therapy using BCI systems will follow patterns observed with traditional therapies or exhibit a different set of dose-response relationships.

Given the heavy emphasis on neuromodulatory training inherent in rehabilitative applications of BCI devices and the brain-behavior relationships that have been observed in individuals receiving these types of therapies (Varkuti et al., 2013; Song et al., 2014; Young et al., 2014b,c), BCI therapies may effect both functional gains and changes in brain activity associated with the impaired function that track or predict these gains in function. Depending on the types of tasks trained, there may also be additional functional improvements with rehabilitative BCI therapy that are not well-reflected by concurrent brain changes. While early findings in the use of BCI therapies for motor rehabilitation after stroke are promising, little to no information is yet available on how dosing parameters for these new therapies may affect behavioral outcomes or brain changes and in what ways the two may be related.

Early neuroimaging studies in stroke survivors receiving rehabilitative therapies using BCI systems have also shown brain changes concurrent with the use of these therapies (Caria et al., 2011; Shindo et al., 2011; Varkuti et al., 2013; Mukaino et al., 2014; Song et al., 2014; Young et al., 2014b,c), and in some cases these markers of neuroplastic reorganization also correlate with individual behavioral gains (Varkuti et al., 2013; Song et al., 2014; Young et al., 2014b,c). One quantifiable measure of neural activity is Laterality Index (LI), which reflects the degree to which a particular function is lateralized between the two hemispheres of the brain. LI can therefore be used as a marker of functional brain organization (Springer et al., 1999; Kundu et al., 2013) and has been applied in studies of stroke rehabilitation to examine relationships between changes in brain activation laterality and behavioral improvements using a variety of newer rehabilitative therapies (Johansen-Berg et al., 2002; Bhasin et al., 2012; Kononen et al., 2012; Orihuela-Espina et al., 2013; Pinter et al., 2013; Yamada et al., 2013) including approaches incorporating BCI technology (Young et al., 2014c).

In this study we examine relationships in therapy administration parameters with changes in motor network LI and with gains in behavioral outcomes in a group of stroke patients with persistent upper extremity motor impairment receiving therapy using a BCI system. With more studies showing increased therapy to be associated with greater improvement among stroke survivors, we hypothesize that individuals receiving higher doses, intensities, and frequencies of BCI therapy will show greater amounts of change in motor network LI as well as greater gains in behavioral measures.

## Materials and Methods

### General Approach

We take advantage of random events and factors that affected the effective dose, intensity, and frequency of therapy administered in the context of an on-going stroke rehabilitation study using BCI therapy to examine how differences in these factors relate to changes observed in brain and behavioral measures assessed at different points relative to therapy administration. Specifically, parameters reflecting therapy dose, therapy intensity, and therapy frequency were analyzed for correlations with changes in LI and with gains in the Action Research Arm Test (ARAT), 9-Hole Peg Test (9-HPT), and Stroke Impact Scale (SIS).

### Subject Recruitment and Study Design

Subjects were recruited as part of an on-going study investigating interventional therapy using a BCI device for stroke rehabilitation targeting upper extremity motor function. This study has been registered with ClinicalTrials.gov and assigned identifier NCT02098265. All subjects were individuals aged 18 years or older who had persistent upper extremity motor impairment as a result of stroke.

Potential subjects were excluded from study participation if they had additional neuropsychiatric diagnoses (e.g., epilepsy, Alzheimer’s, schizophrenia) or if they were allergic to electrode gel, tape, or metal against the skin. Potential subjects were also excluded if they were receiving treatment for any infectious diseases, if they had any apparent oral lesions or active inflammation of the oral cavity, if they were pregnant or likely to become pregnant during the course of study participation, if they had any contraindications for MRI, or if they were unable to provide informed consent. This study was approved by the University of Wisconsin Health Sciences Institutional Review Board. All subjects provided written informed consent upon enrollment.

This set of analyses uses a retrospective, single-group design with four assessment points, taking advantage of variations in BCI therapy administration parameters that arose due to differences in subject preferences and random factors.

### Intervention Schedule and Behavioral Assessments

All subjects were scheduled to receive at least 9 and up to 15 2-h sessions of BCI therapy, with up to 3 sessions per week and no more than one session occurring on the same day. In general, subjects were scheduled for the maximum 15 sessions, although many did not complete all 15 due to factors such as illness, extreme weather conditions, or technical difficulties. In some cases, make-up sessions were arranged upon the cancellation of one or more sessions depending on staff availability as well as on subject willingness and availability.

Subjects were assessed at four time points relative to the administration of BCI therapy: pre-therapy (no more than 1 week before the first BCI therapy session), mid-therapy (after completion of at least 5 BCI therapy sessions), post-therapy (within 1 week after completing the last BCI therapy session), and 1 month after completion of all BCI therapy. Behavioral assessments and MRI scans were obtained on each assessment day.

Behavioral measures included the ARAT (Carroll, 1965; Lang et al., 2006), the 9-HPT (Beebe and Lang, 2009), and the SIS (Duncan et al., 1999; Carod-Artal et al., 2008) and were evaluated at each of the four assessment visits. Total ARAT scores for the subject’s impaired hand were examined for this study. Scores for the 9-HPT were calculated as the average time (in seconds) needed to complete the task between two attempts both using the impaired hand. This study focused on the Activities of Daily Living (SIS ADL), Hand Function (SIS HF), and Strength (SIS Strength) domains of the SIS, as the SIS ADL was the domain most reflective of global function, the SIS HF domain was the one most closely related to motor functions practiced during BCI therapy, and the SIS Strength was a second domain reflecting more general motor function. In accordance with standard SIS scoring practice, SIS domain scores were transformed independently to reflect the percent possible points obtained by each subject for each domain.

### MRI Acquisition and Processing

Both anatomical and functional MRI scans were obtained at each assessment. MRI scans were obtained for all subjects on one of three 3-Tesla GE MR750 scanners equipped with high-speed gradients (Sigma GE Healthcare, Milwaukee, WI, USA) using an 8-channel head coil. Scanning parameters for the T1-weighted anatomical images were: field of view 246 mm, matrix 256 × 256, TR 8.16 ms, TE 3.18 ms, flip angle 12°, constituting a BRAVO FSPGR pulse sequence. Each anatomical image comprised 156 axial plane slices of thickness 1 mm and 1 mm spacing between slices. Scanning parameters for fMRI scans were: field of view 224 mm, matrix size 64 × 64, TR 2.6 s, TE 22 ms, flip angle 60°, constituting a T2^∗^-weighted gradient-echo echo-planar imaging pulse sequence sensitive to BOLD contrast. Functional MRI scans comprised 70 sequential whole-brain acquisitions of 40 axial plane slices acquired with no spacing between adjacent slices using interleaved acquisition. This yielded 3.5 mm isotropic resolution. Padding was used around each subject’s head to help minimize movement, and subjects were instructed to keep their head still during the scan period.

Unless otherwise noted, all pre- and post-processing of anatomical and functional MRI scans for this study was completed using the Analysis of Functional NeuroImaging (AFNI) software package (Cox, 1996). For each functional sequence, the first three volumes were discarded to allow for signal stabilization before the data sets were corrected for motion and spatially smoothed using a 6 mm FWHM Gaussian kernel. Each voxel time series was then scaled to a mean of 100, and a voxel-wise regression analysis was used to regress out each of six motion parameters. This process yielded maps of a voxel-wise *t*-statistic onto which AFNI’s 3dClustSim function was applied to identify minimum cluster sizes needed for cluster-wise correction of multiple comparisons at a significance level of *p* < 0.05.

Skull-stripped anatomical and EPI data sets were visually checked for alignment and for appropriate skull stripping of the anatomical image. In cases where automated skull stripping was too aggressive around the area of stroke lesion, skull stripping was instead performed using different options in FSL’s (FMRIB Software Library v. 5.0; Smith et al., 2004) BET (Brain Extraction Tool; Smith, 2002), and align_epi_anat.py was used to align any anatomical and EPI data sets for which alignment was not adequate. Skull stripped, aligned anatomical brain images were then used for subsequent transformations between subject space and Talairach space (Talairach and Tournoux, 1988).

Given the motor-oriented emphasis of the rehabilitative intervention and the fact that this BCI therapy is designed to provide feedback on neuromodulation in areas of the motor and premotor cortex, we focus the neuroimaging analyses in this paper to LI values as calculated from activity in the motor network. LI values were calculated in a manner consistent with previously described methods (Young et al., 2014c). In brief, a mask for each side of the motor network was constructed based on motor network regions previously identified from an independent component analysis of whole-brain resting state fMRI scans in a cohort of healthy normal subjects (Shirer et al., 2012). These regions included primary motor, premotor, thalamic, and cerebellar areas and were consistent with areas classically identified as important in facilitating coordinated motor movements. The full set of functional brain networks identified from this independent component analysis are freely downloadable at http://findlab.stanford.edu/functional_ROIs.html (Shirer et al., 2012). Cluster-wise correction for multiple comparisons was applied to each activation map obtained from stroke subjects in the present study using the minimum cluster sizes estimated for each mask from 3dClustSim. Voxel counts were obtained by calculating the number of voxels surviving within the mask for each side of the motor network when thresholded at a significance level of *p* < 0.0001. LI was then calculated using the formula (V_I_-V_C_)/(V_I_+V_C_), where V_I_ is the number of voxels in the ipsilesional hemisphere mask with significant activation at the preset statistical threshold and V_C_ is the number of voxels in the contralesional hemisphere mask with significant activation at the same threshold (Springer et al., 1999). Using this formula, more negative LI values reflect greater activation in the contralesional hemisphere while more positive LI values reflect greater activation in the ipsilesional hemisphere. This yielded a quantitative measure of brain activity lateralization during finger tapping of the impaired hand. These calculations were also performed for two additional mask sets – a mask set encompassing the whole brain and a mask set comprising only the cortical areas of the motor network masks used in the main analyses – at the same significance level (*p* < 0.001) as well as at a less stringent significance level (*p* < 0.05). A listing of the anatomical components used in creating each of these mask sets can be found in the supplemental materials. The results from analyses using these two additional mask sets are also presented in the supplement materials but have been excluded from the main analyses reported here, as they do not directly contribute to the investigation of our main hypothesis focused on the motor network.

### MRI Task Instructions

Each 3-min fMRI scan consisted of nine 20-s blocks that alternated between the rest (five blocks total) and tap (four blocks total) conditions. Subjects were given a button box in their impaired hand and instructed to tap the buttons on the box using the second through fifth fingers of the impaired hand sequentially and continuously during blocks of tapping and to relax their hand and rest during blocks of rest. This self-paced tapping was cued using visual cues on a slide show that displayed the word “Rest” during blocks of rest and the word “Tap” during blocks of tapping. Two subjects whose vision could not be sufficiently corrected or accommodated in the MRI to adequately view the instructions on the slideshow were given tactile cues in the form of a single tap on the leg at the beginning of each block in order to cue when to alternate between blocks of tapping and blocks of rest. Subjects who were unable to produce detectable finger tapping movements using the button box were assisted by a researcher during the scan to perform assisted finger tapping of the impaired hand. This assistance was provided in the form of a member of the research team standing in the scanner room during acquisition and moving the fingers of the impaired hand up and down in a similar “button-pressing” motion sequentially and continuously during blocks of tapping and leaving the subject’s hand still to rest during blocks of rest. Assisted movements were performed at a rate of approximately one assisted finger “press” per second, with flexibility to accommodate individual subject comfort and degree of spasticity in the impaired hand. Nine subjects received this type of assistance.

### BCI Therapy and Session Sequence

The BCI system and therapy sequence were consistent with those previously described (Young et al., 2014a,b,c), using BCI 2000 software (Schalk et al., 2004) version 2 with in-house modifications for input from a 16-channel EEG cap and amplifier (Guger Technologies) and integration with tongue stimulation (TDU 01.30 Wicab Inc.) and functional electrical stimulation (FES) (LG-7500, LGMedSupply; Arduino 1.0.4). In short, an open-loop screening task at the beginning of each session presented each subject with repeated, randomly ordered 4-s visual cues of “Left,” “Right,” or “Rest,” during which the subject was instructed to perform attempted movement of the corresponding hand or to rest. EEG activity recorded during this open-loop screening task was then used to determine the optimal control signals as previously described (Wilson et al., 2009). Movements practiced during BCI therapy sessions varied among subjects based on subject preference and the baseline abilities and recovery goals of each individual. Signals focused on the Mu (8–14 Hz) and Beta (18–26 Hz) frequency ranges detected by EEG over the motor cortex. All movements involved repeated attempts at motion in the hand or wrist. Opening and closing of the hand and wrist extension were two common motions that subjects elected to practice during BCI therapy.

After appropriate control signals had been identified, subjects were taught to perform a closed-loop task. For the closed-loop task, subjects were instructed to maneuver an on-screen cursor to a target also presented on-screen located on either the right or left side of the screen. Subjects were instructed to use attempted movements of the right or left hand to drive the cursor to the right or left side of the screen respectively as appropriate for the target presented during each trial. Lateral cursor movement was determined by real-time EEG signals based on control signals determined from data acquired during the open-loop screening task. Trials were grouped in runs, with one run comprising 8–12 individual trials with one target presented during each trial. This task was first performed with no external stimuli (i.e., visual feedback only) and then performed with the addition of triggered FES and tongue stimulation. Subjects were encouraged to complete at least 10 runs without external stimulus and then at least 10 runs with external stimuli, time permitting. No upper limit was specified on the number of runs that could be delivered during a given session.

Subjects were also offered the opportunity to take a break at each transition before beginning a new task or stimulus and were told that they were also allowed to take breaks any time between runs upon request.

### Determination of Therapy Parameters

Therapy dose was calculated in two ways. The first was as the total number of 2-h BCI therapy sessions that the subject had completed at the time of a given assessment (i.e., “therapy sessions dose”). The second was as the total number of BCI runs completed summed across all BCI therapy sessions that the subject had completed at the time of a given assessment (i.e., “therapy runs dose”). Therapy intensity was calculated as an average number of runs completed per session averaged across all BCI therapy sessions completed up to the point of assessment. Therapy frequency was calculated as the ratio of total sessions completed divided by the total days that had passed since the day of baseline assessment, and subjects were binned as either “low” (an average of ≤2 therapy sessions per week) or “high” (an average of >2 therapy sessions per week) therapy frequency.

### Statistical Analysis

All statistical analyses for this study were performed using R statistical software (version 3.0.1). Changes from baseline group averages in impaired hand ARAT scores and in scores for each SIS domain examined were compared to the corresponding estimated values for the minimum clinically important difference (MCID) in chronic stroke for these measures (van der Lee et al., 2001a; Lin et al., 2010). As there are no established MCID values for 9-HPT times in chronic stroke, linear mixed-effect modeling was used to analyze group 9-HPT scores collected at each assessment for differences relative to pre-therapy baseline values. Linear mixed-effect modeling was also used to analyze group LI values collected at each assessment for differences relative to pre-therapy baseline values.

Generalized estimating equations (GEEs; Ballinger, 2004) were used to examine correlations between changes in behavioral measures from baseline pre-therapy values with therapy sessions dose, therapy runs dose, and therapy intensity. This approach was also used to examine correlations between changes in LI from baseline pre-therapy values with these same three therapy parameters. A GEE approach was also used to investigate brain-behavior relationships, examining correlations between changes in LI values with changes in behavioral measures.

Linear mixed-effect models were used to assess for any effect of low vs. high therapy frequency on changes in LI values or in behavioral measures from pre-therapy baseline values at the mid-therapy and post-therapy assessments.

These approaches were chosen in order to allow for data obtained from the same subjects over multiple assessments to be incorporated into the same models, as they are able to accommodate repeated measures designs and do not make the assumption of independence among all data points as more traditional statistical approaches often require. These approaches were also chosen because they are better able to accommodate missing data points from cases in which individual subjects did not complete all four assessment sessions.

All *p*-values generated from these analyses were corrected for multiple comparisons using false discovery rate (fdr) correction, adjusting raw *p*-values to yield adjusted *p*-values following the Benjamini and Hochberg (1995) method. Correction using the fdr method was chosen because it is a commonly accepted approach to accounting for multiple comparisons when family wise error rate minimization may be too strict (Noble, 2009), and fdr has been suggested as a more appropriate method for *p*-value correction in health studies than the more traditional yet more conservative Bonferroni correction (Glickman et al., 2014).

In order to avoid biasing analyses with data from floor and ceiling effects, subjects who displayed floor or ceiling effects on a given behavioral measure (i.e., consistently scoring the absolute minimum or absolute maximum for that measure pre-therapy as well as on all subsequent assessment days) were excluded from all analyses using data from that outcome measure. Thresholds for significance and trend toward significance were set *a priori* at *p* ≤ 0.05 and 0.05 < *p* < 0.1 respectively for all statistical analyses described. Adjusted *p*-values were compared to these thresholds.

## Results

### Participant Characteristics and Retention

Sixteen individuals with a history of stroke resulting in persistent upper extremity impairment were used for the analyses presented in this report. These subjects were all enrolled into the study as previously described and comprise the first 16 subjects enrolled in the study following adult-onset stroke who had been assessed at least through the mid-therapy time point and were not scheduled to receive any additional therapy or assessments at the time of these analyses.

Subjects in this cohort comprised a predominantly right-handed cohort, with one subject (Subject 13) being left-handed, another (Subject 5) being ambidextrous, and the remaining 14 subjects being right-handed as determined using the Edinburgh Handedness Inventory (Oldfield, 1971). A summary of further subject characteristics is provided in **Table 1**.

**Table 1:** Participant characteristics.

Subject	Sex	Age (years)	Stroke location	Impaired hand	NIHSS	Months from stroke
1	M	52	L MCA	R	8	15
2	F	61	L frontal lobe	R	8	16
3	M	68	L frontal lobe	R	0	3
4	M	66	L MCA	R	6	23
5	F	73	L MCA	R	0	2
6	M	59	L pontomedullary junction	R	4	144
7	M	59	L MCA	R	2	28
8	F	45	R MCA	L	6	99
9	F	71	R MCA	L	6	6
10	M	80	R occipital lobe	L	2	20
11	F	75	R putamen	L	7	23
12	M	61	L basal ganglia	R	0	17
13	M	48	R pons	L	3	6
14	M	59	L MCA	R	2	28
15	M	48	R medulla	L	6	5
16	M	50	R MCA	L	4	16

M, male; F, female; L, left; R, right; MCA, middle cerebral artery; NIHSS, National Institutes of Health Stroke Scale.

Of these 16 subjects, 14 completed all scheduled assessments. None of the subjects who participated reported any adverse events or problems with using the BCI device, but two did fail to complete all four planned assessment visits. One subject did not complete the final assessment due to scheduling incompatibilities between the subject’s availability and the availability of the MRI scanners during the appropriate 1 month post-therapy time window. Another subject withdrew from the study after completing the mid-therapy assessment because the subject could no longer afford transportation to and from the study site.

All subjects completed at least five BCI therapy sessions before mid-therapy assessment and between 9 and 15 BCI therapy sessions before post-therapy assessment. All subjects completed at least 50 runs of BCI training by the mid-therapy assessment point and at least 124 runs of BCI training over the course of the entire therapy period. A summary of the effective BCI therapy administration parameters for the group is presented in **Table 2**.

**Table 2:** Effective group BCI therapy administration parameters.

	Total sessions	Total runs	Runs/session
Mid-therapy assessment	6.43 (1.50)	164.81 (66.83)	25.33 (8.09)
Post-therapy assessment	13.40 (2.41)	379.60 (154.95)	27.44 (8.29)

Numbers shown in X (Y) format represent mean (standard deviation) and reflect total cumulative counts or averages for each parameter at the time of the indicated assessment.

At mid-therapy assessment, seven subjects were classified as “low” therapy frequency and nine subjects were classified as“high” therapy frequency. At post-therapy assessment, seven subjects were classified as “low” therapy frequency and eight subjects were classified as “high” therapy frequency.

It was informally observed that a number of factors could influence the number of BCI therapy sessions and runs completed. Factors related to subject characteristics that influenced these parameters were often related to scheduling. In particular, it was observed that most subjects were not able to drive themselves to and from the study center and therefore the frequency of therapy sessions often depended greatly on the availability of the subjects’ transportation arrangements to and from study sessions. Subject health also influenced attendance at scheduled BCI therapy sessions, with unanticipated illnesses (e.g., flu, pneumonia) leading to one or more canceled sessions for at least two subjects. The number of breaks each subject requested during BCI therapy sessions varied among subjects but was not explicitly quantified. It was also noted that subjects who lived further from the study center (e.g., >2 h driving distance each way) tended to request twice-per-week therapy sessions rather than thrice-per-week therapy sessions. Some subjects were also more interested than others in arranging additional make-up sessions after one or more scheduled sessions had been canceled.

Factors not related to subject characteristics that influenced the number of BCI therapy sessions and runs completed included extreme weather (e.g., therapy sessions were canceled when weather conditions made it unsafe to travel to and from the study center), technical difficulties or isolated equipment malfunction, and the occurrence of major holidays during the period of subject participation (e.g., a subject who normally receives therapy on a Monday-Wednesday-Friday schedule may only be scheduled for Monday and Wednesday the week of the Thanksgiving holiday). Availability of the MRI scanner was also a factor, with occasional limitations on when MRI scans could be scheduled, influencing how many BCI therapy sessions could be completed before each MRI scan. Availability of the MRI scanner as well as availability of research staff also affected whether or not additional make-up sessions could be arranged after one or more scheduled sessions were canceled due to various factors as described above.

### Neuroimaging Outcome Measures

**FIGURE 1:**
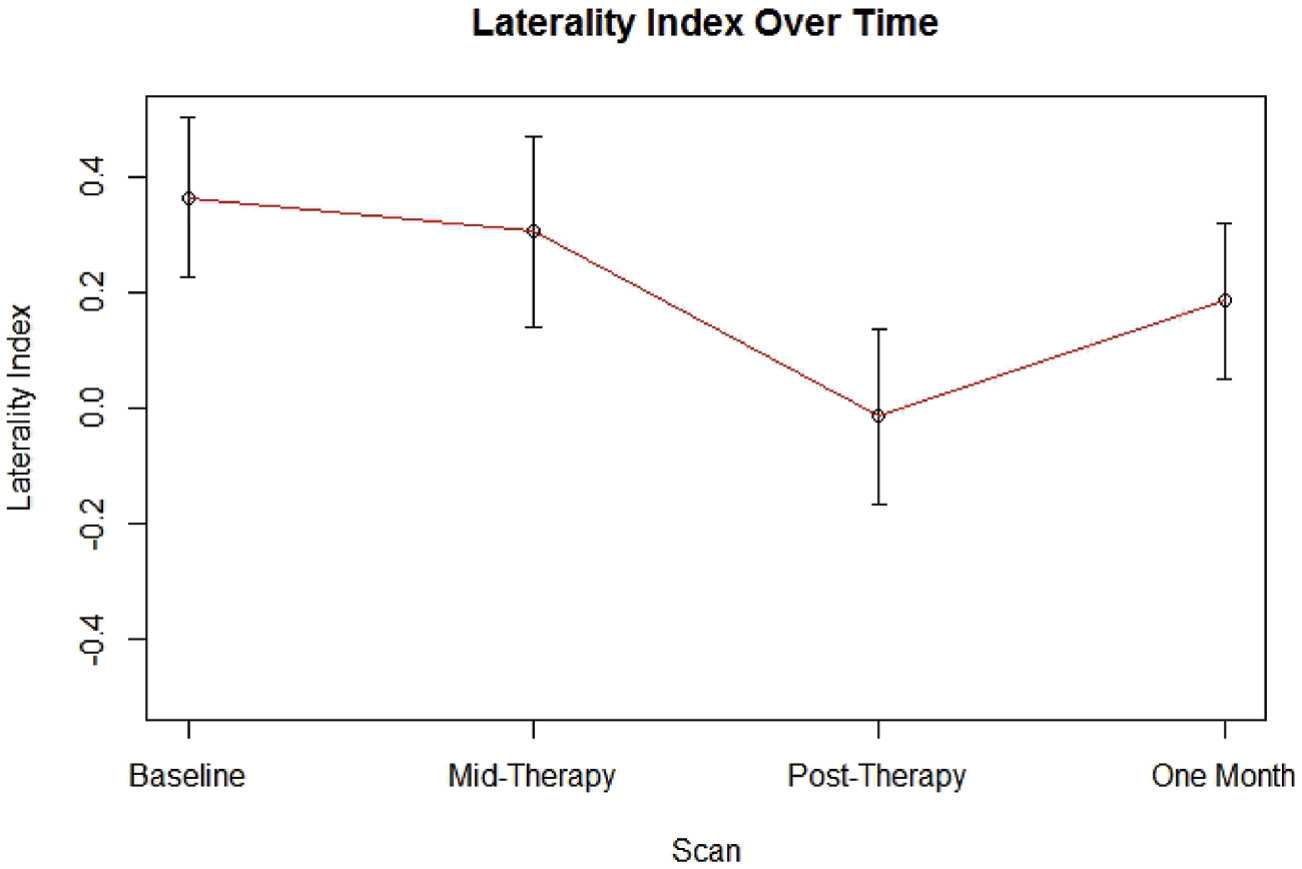
**Average LI values at each scan time point.** Error bars represent standard error.

**FIGURE 2:**
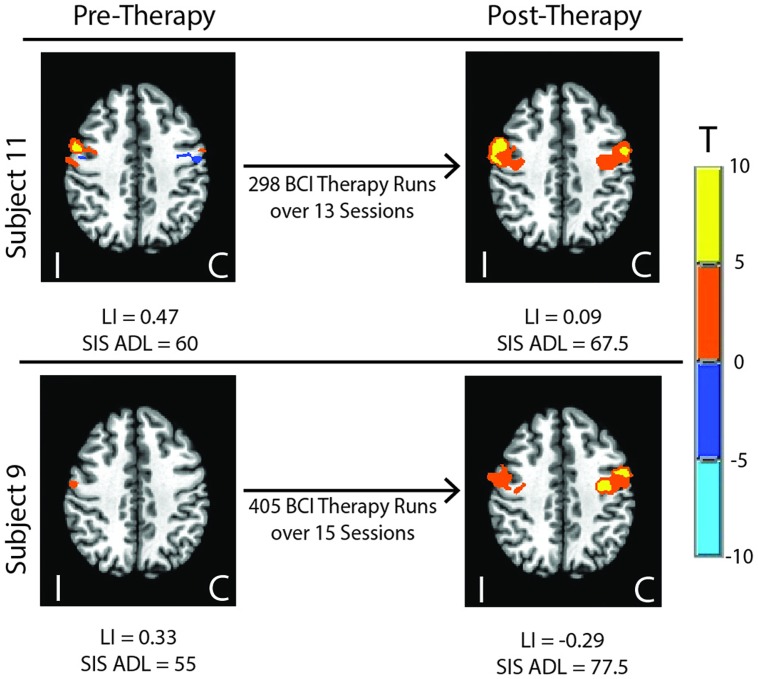
**Sample fMRI images showing progression from pre-therapy to post-therapy activation within motor network areas.** Colored regions show areas masked within the motor network with significant activation at *p* ≤ 0.05. LI, Laterality Index; SIS, Stroke Impact Scale; ADL, Activities of Daily Living; I, ipsilesional; C, contralesional.

Group LI outcome measures at each time point are presented in **Figure 1**. An analysis using linear mixed effect modeling found no significant effect from baseline in LI values at the group level at any subsequent time point (*p* > 0.05, df = 36). Linear mixed-effect modeling also found no significant differences in LI values recorded between subjects who performed independent finger tapping of the impaired hand and subjects who were assisted with making finger tapping motions of their impaired hand (*p* > 0.05, df = 14). Examination of the LI values obtained from the two subjects who were not right-handed were not found to be outliers among the LI data collected from the cohort at any time point. Sample fMRI images showing progression from pre-therapy to post-therapy time points are provided in **Figure 2**.

### Behavioral Outcome Measures

**FIGURE 3:**
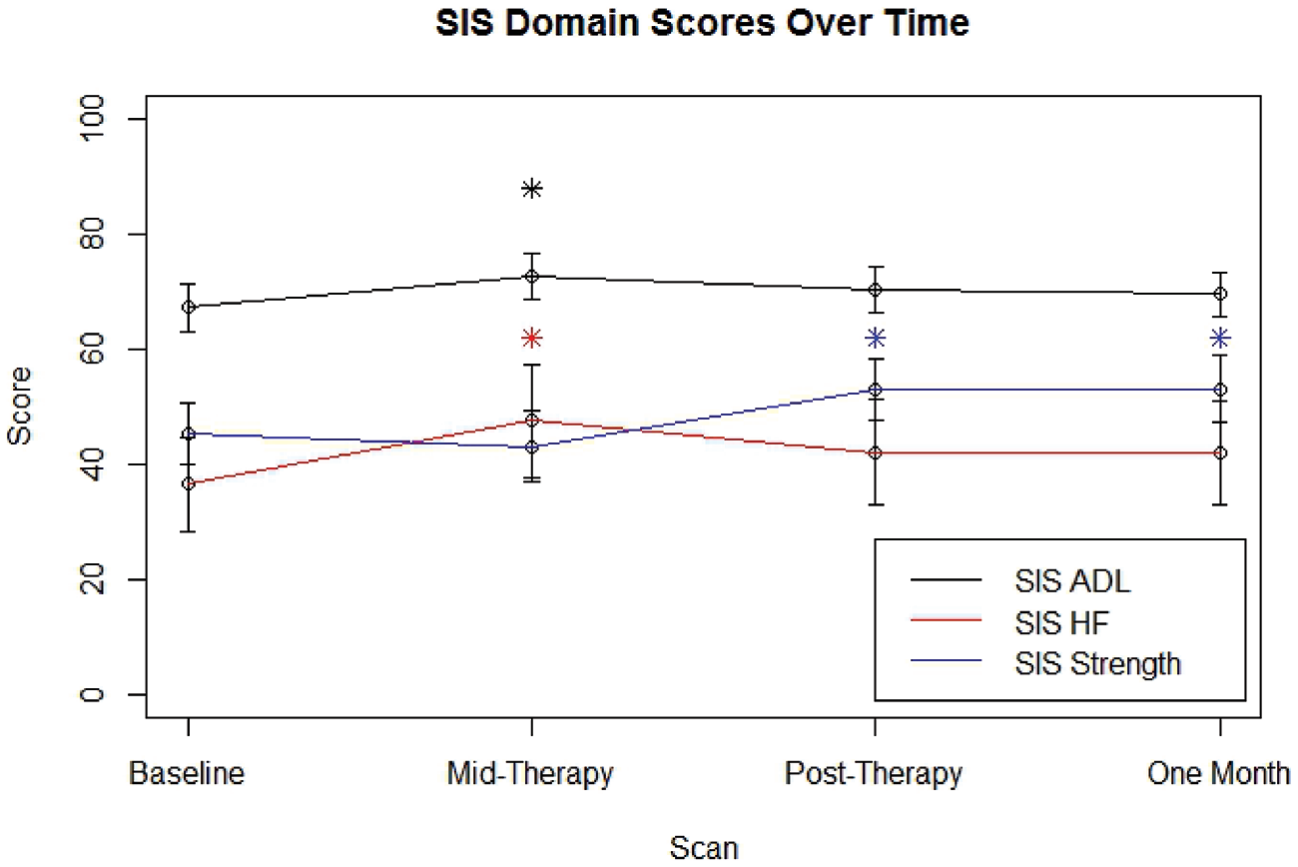
**Average SIS scores for each domain examined at each scan time point.** Error bars represent standard error. SIS, Stroke Impact Scale; ADL, Activities of Daily Living; HF, Hand Function. ^∗^Improvement over baseline values meeting distribution-based MCID and also falling within the 95% confidence interval for meeting anchor-based MCID.

After removing the six individuals who displayed floor or ceiling effects in SIS HF scores, group SIS performance for each domain examined at each time point is shown in **Figure 3**. Group-level improvements from baseline SIS scores that met the distribution-based MCID estimates that were also large enough to fall into the 95% confidence interval of anchor-based MCID estimates were noted at mid-therapy for SIS ADL (*n* = 16) and SIS HF (*n* = 10) scores and were also observed post-therapy and at 1 month follow-up for SIS Strength (*n* = 16) scores.

After removing the four individuals who displayed floor or ceiling effects on the ARAT, group ARAT performance (*n* = 12) did not meet the MCID of an improvement of six points above baseline at any assessment time point. Linear mixed-effect model analysis of 9-HPT times among the six subjects able to perform the 9-HPT did not reveal any significant group-level changes from baseline in 9-HPT performance (*p* > 0.05, df = 14).

### BCI Therapy Dosing Parameter Correlations with Changes in Behavioral Measures

**FIGURE 4:**
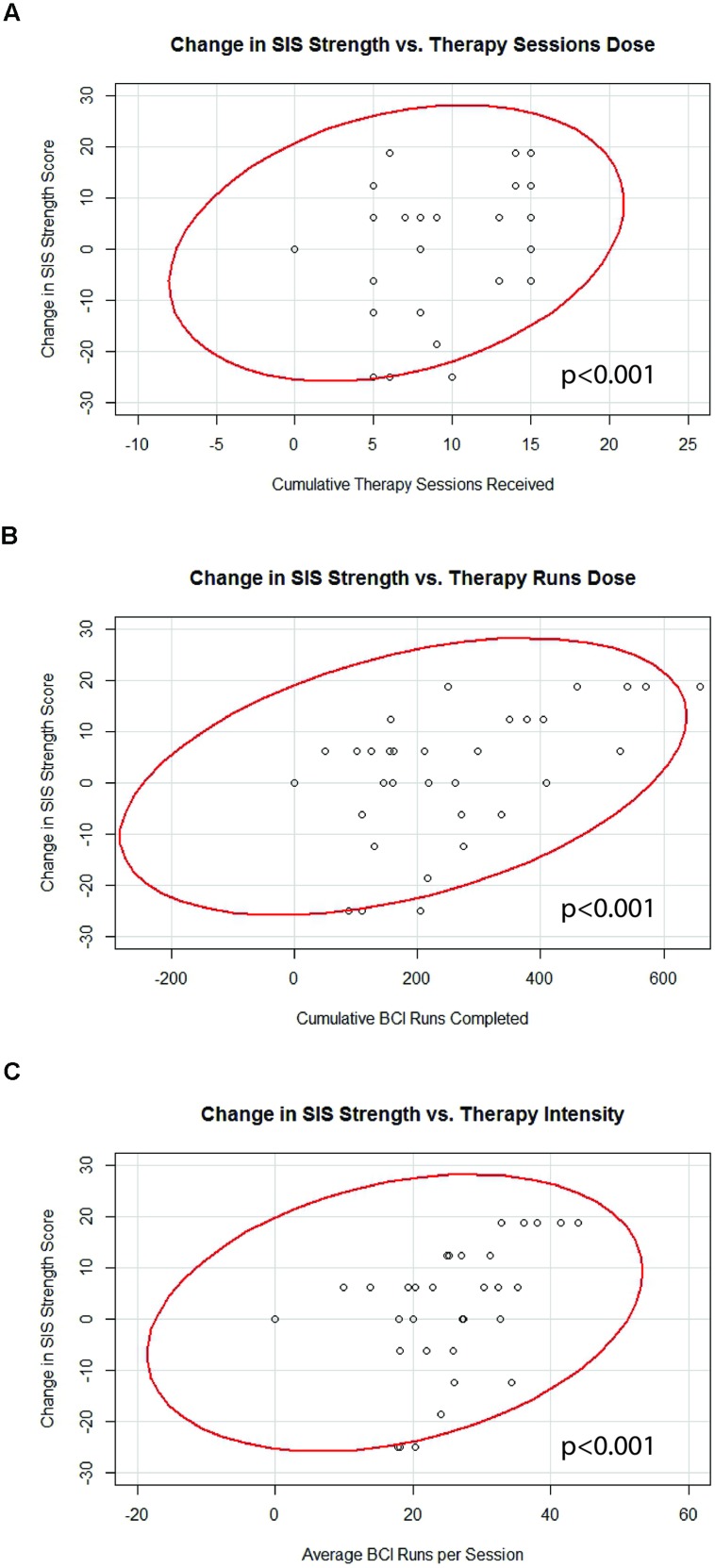
**Significant correlations identified between changes in SIS Strength scores and **(A)** therapy sessions dose, **(B)** therapy runs dose, and **(C)** therapy intensity.** Red lines represent data ellipses at the 95% confidence level. SIS, Stroke Impact Scale; BCI, brain-computer interface.

Generalized estimating equation analysis identified significant correlations between changes in SIS Strength and the three therapy parameters of therapy sessions dose, therapy runs dose, and therapy intensity. These correlations are summarized in **Table 3** and shown in **Figure 4**. No significant correlations were identified between changes in SIS ADL, changes in SIS HF, changes in ARAT scores, or changes in 9-HPT performance with any of the therapy parameters of therapy sessions dose, therapy runs dose, or therapy intensity. Linear mixed-effects analysis did not identify any significant differences between “low” vs. “high” therapy frequency on changes in scores for SIS Strength (df = 14), SIS ADL (df = 14), SIS HF (df = 9), ARAT (df = 10), or 9-HPT (df = 5; *p* > 0.05 for all “low” vs. “high” therapy frequency and behavioral change analyses).

**Table 3:** Correlations between BCI therapy parameters and changes in Stroke Impact Scale Strength scores.

BCI therapy parameter	Estimated β	Wald coefficient	*p*-value
Therapy sessions dose	0.828	15.804	*p* < 0.001^∗^
Therapy runs dose	0.028	41.469	*p* < 0.001^∗^
Therapy intensity	0.290	14.431	*p* < 0.001^∗^

*p*-values shown after correction for multiple comparisons using fdr correction. *Significant at *p* ≤ 0.05.

### BCI Therapy Dosing Parameter Correlations with Brain Changes (fMRI-LI)

**FIGURE 5:**
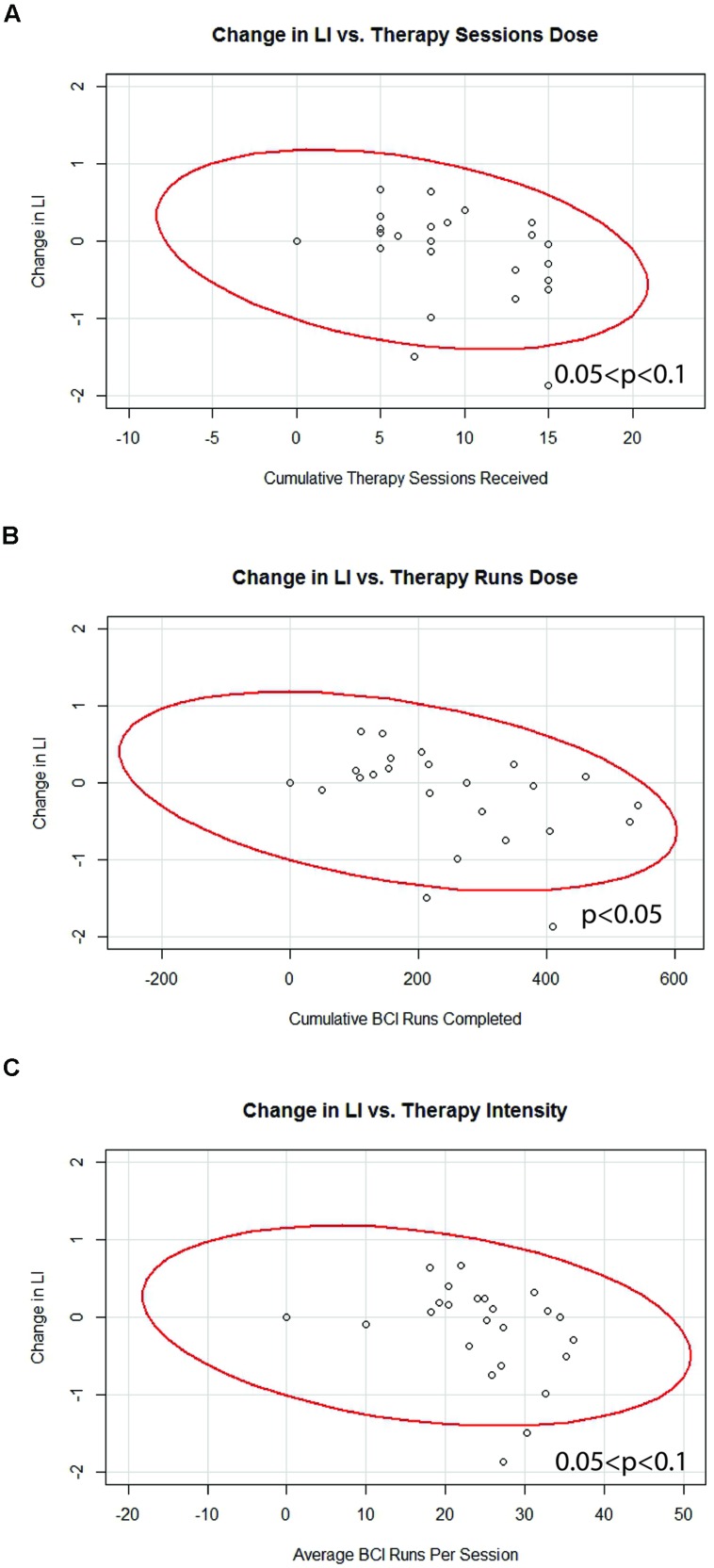
**Significant correlations identified between changes in motor network LI and **(A)** therapy sessions dose, **(B)** therapy runs dose, and **(C)** therapy intensity.** Red lines represent data ellipses at the 95% confidence level. LI, Laterality Index; BCI, brain-computer interface.

Generalized estimating equation analysis showed significant correlations between changes in LI values and therapy runs dose, while correlations that trended toward significance were also identified between changes in LI values and therapy sessions dose as well as therapy intensity. These relationships are summarized in **Table 4** and shown in **Figure 5**. No significant effects were identified when assessing for differences between “low” vs. “high” therapy frequency on changes in LI values using a linear mixed-effects analysis.

**Table 4:** Correlations between BCI therapy parameters and changes in laterality index.

BCI therapy parameter	Estimated β	Wald coefficient	*p*-value
Therapy sessions dose	-0.032	4.969	0.074^+^
Therapy runs dose	-0.001	7.130	0.029^∗^
Therapy intensity	-0.011	5.309	0.070^+^

*p*-values shown after correction for multiple comparisons using fdr correction. ^∗^Significant at *p* ≤ 0.05. ^+^Trend toward significance at 0.05 < *p* < 0.1.

### Brain Change (fMRI-LI) Correlations with Changes in Behavioral Measures

Generalized estimating equation analysis identified significant correlations between changes in LI values and changes in scores for SIS ADL and changes in 9-HPT performance. These relationships are summarized in **Table 5**. No significant correlations were identified between changes in SIS Strength scores, SIS Hand Function scores, or ARAT scores with changes in LI.

**Table 5:** Correlations between changes in laterality index and changes in behavioral scores.

Behavioral measure	Estimated β	Wald coefficient	*p*-value
SIS ADL	-4.681	9.202	0.011^∗^
9-HPT	18.253	17.755	*p* < 0.001^∗^

SIS, Stroke Impact Scale; ADL, Activities of Daily Living; 9-HPT, 9-Hole Peg Test.

*p*-values shown after correction for multiple comparisons using fdr. ^∗^Significant at *p* ≤ 0.05.

For additional analysis and results obtained from LI calculations using other masks and varied thresholds, the interested reader is referred to the supplementary materials.

## Discussion

Although stroke rehabilitation is a growing area of research, there have been relatively few studies examining dose-response relationships for treatments aimed at facilitating stroke recovery. In fact, knowledge of optimal rehabilitative dosing parameters is sparse enough within the area of any given modality that official guidelines do not make specific recommendations regarding optimal intensity or duration of rehabilitative treatments after stroke (Duncan et al., 2005). To our knowledge, this is the first examination of dose-response relationships relating therapy administration parameters to brain and behavioral outcomes using a BCI system for stroke rehabilitation. These preliminary findings of increased therapy sessions, increased BCI runs, and increase therapy intensity correlating with better outcomes in SIS Strength (**Table 3**; **Figure 4**) are consistent with the relatively common pattern established among traditional rehabilitative therapies where better outcomes are associated with increased therapy (Nugent et al., 1994; Langhorne et al., 1996; Cifu and Stewart, 1999; Kwakkel et al., 1999; van der Lee et al., 2001b; Haines et al., 2011).

The finding relating increases in SIS Strength scores to increases in BCI therapy is also consistent with other work that has begun investigating similar dose-response relationships in newer, non-traditional therapy modalities. One meta-analysis that combined study results across both traditional and emerging stimulus-free rehabilitation therapies for stroke found increased therapy time to be associated with better outcomes (Lohse et al., 2014). Similarly, some studies specific to robot-assisted and transcranial direct current stimulation approaches also conform to this general trend, revealing that higher therapy intensity and dose may be needed in order for significant improvements over control outcomes to be achieved (Burgar et al., 2011; Hsieh et al., 2012; Feng et al., 2015).

However, no other behavioral outcome measures examined in this set of analyses were found to have direct relationships with BCI therapy administration parameters. This absence of a direct dose-response relationship among other behavioral outcome measures is more consistent with some of the findings among newer therapy modalities showing no additional improvement or even less improvement with increased therapy administration. One randomized controlled trial examining dose-response relationships with CIMT found that patients receiving high-dose CIMT actually showed significantly less improvement than those with low-dose CIMT or traditional control therapy (Dromerick et al., 2009). Similarly, some studies using therapeutic approaches such as mobilization and tactile stimulation (Hunter et al., 2011) or neuromuscular electrical stimulation (Hsu et al., 2010) find no statistically significant effects of therapy parameter modification.

The incorporation of brain-based neuroimaging metrics in this set of dose-responses analyses may help explain why some behavioral outcome measures appear to respond to differences in BCI therapy administration parameters while others do not. In this study, therapy dose and therapy intensity reached significance or a trend toward significance in correlation with changes in motor network LI (**Table 4**; **Figure 5**). These correlations were consistently negative, with increases in BCI therapy corresponding to greater recruitment of contralesional motor network areas, as reflected in more negative changes in LI values. The association between additional BCI therapy and greater activation in contralesional motor network areas is consistent with previous preliminary work from our group that has documented greater contralateral brain activation over the course of BCI therapy and that has shown such decreases in LI to be associated with increased functional recovery in a small but similar cohort using BCI-based therapy for stroke rehabilitation (Young et al., 2014c). Relationships between changes in LI values and changes in behavioral outcome measures were also observed in the present set of analyses (**Table 5**), with greater contralesional motor network recruitment again being associated with improvements in functional outcomes.

The exact mechanism by which more negative LI values and therefore increased recruitment of contralesional motor network areas are associated with improvements in behavioral outcomes using this BCI therapy remains unclear. Previous studies relating changes in LI to motor outcomes after BCI therapy have tended to find the opposite pattern in which greater lateralization of brain activity to the ipsilesional hemisphere is associated with improved functional performance (Caria et al., 2011; Ramos-Murguialday et al., 2013). However, these studies were based on samples largely restricted to subcortical stroke patients who by definition have sustained minimal to no direct cortical damage. In contrast, participants in the present study have comprised mostly subjects with cortical stroke, often with relatively large areas of cortical infarct. Given this key difference, greater recruitment of the contralesional hemisphere may be necessary to facilitate functional improvements after stroke in the presence of more extensive cortical damage (Stinear et al., 2007; Schlaug et al., 2011; Di Pino et al., 2014), which may help to explain the pattern observed among the individuals in this study.

Even among individuals suffering from subcortical stroke, coordination with the contralesional hemisphere is still beneficial to severely impaired individuals (Lotze et al., 2006), and one study of a cohort comprising mostly subcortical stroke patients receiving gesture therapy showed behavioral gains concurrent with increased activation of the contralesional motor cortex (Orihuela-Espina et al., 2013). There is further evidence that the specific nature and extent of damage to the corticospinal (Ward et al., 2007) and corticofugal (Newton et al., 2006) fiber tracts affects the subsequent patterns and relationships between brain activity and motor function. Another study examining individuals with heterogeneous stroke locations (including a number of individuals with large cortical strokes) observed that individuals with greater damage to the corticospinal system showed force-related signal changes in the contralesional hemisphere rather than the ipsilesional hemisphere as was observed in control subjects and stroke patients with less corticospinal damage (Schaechter et al., 2008). A similar trend has also been observed across studies examining the cortical effects of FES treatment on stroke patients, with more severely impaired individuals often recruiting from the contralesional hemisphere while less impaired individuals tended to recruit ipsilesional areas (Quandt and Hummel, 2014). Future work will benefit from subanalyses that examine differential dose-response and overall lateralization effects among various subpopulations of stroke subjects receiving BCI-based rehabilitative therapies.

The findings presented in this early analysis suggest differential sensitivities to therapy administration parameters with this BCI system among the outcomes assessed. In particular the relationships identified between changes in LI measures and differences in BCI therapy administration parameters support a model in which neuroimaging measures may be more sensitive or may respond more quickly to changes in BCI therapy dosing parameters than behavioral assessments in which improved performance is associated with concurrently detectable brain changes. For example, although this study did not identify significant correlations between BCI therapy parameters and changes in SIS ADL, improvements in SIS ADL were noted at the group level mid-therapy, and individual gains in SIS ADL did show a relationship with LI changes. In light of the small to moderate sample size, we did not directly test the role of brain changes in mediating the relationship between therapy parameters and behavior. Future work with larger sample sizes will allow for more formal mediation analyses to further investigate the role of brain changes in the effect of BCI therapy on behavioral outcomes.

**FIGURE 6:**
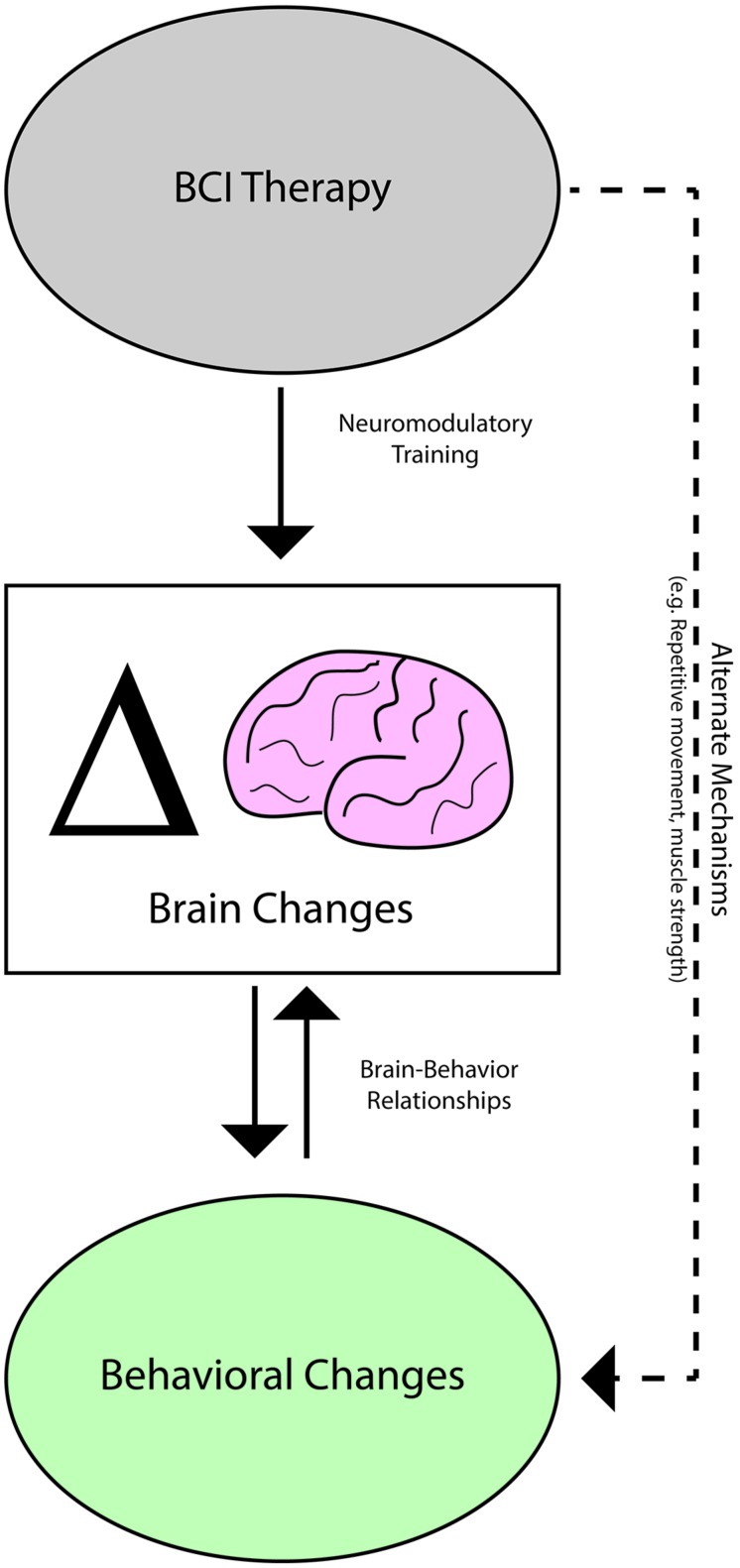
**Potential mechanisms mediating brain activity and behavioral changes observed with BCI therapy**.

The relationships identified in this and in previous studies between neuroimaging measures and behavioral outcomes with rehabilitative BCI therapy after stroke (Varkuti et al., 2013; Song et al., 2014; Young et al., 2014b,c) in combination with the response of LI changes to differences in BCI therapy administration support a model in which some behavioral changes achieved with BCI therapy are associated with concurrent brain changes (**Figure 6**). Given such brain-behavior relationships, brain activity changes as reflected in measures such as LI appear to be a better predictor of behavioral outcomes than therapy dose. Thus, for behavioral outcomes such as SIS ADL and 9-HPT that displayed relationships with LI changes but not with BCI therapy parameters, it may be that the effective dose of therapy as reflected by brain activity measures constitutes a better predictor of behavioral change than administered dose.

In considering the brain-dependent vs. brain-independent pathways by which BCI therapy may be effecting changes in behavioral scores, the finding that improvements in SIS Strength correlated positively with increases in BCI therapy dose and intensity but not with changes in LI values suggests that gains in SIS Strength may be independent of neuroplastic mechanisms. For example, repeated application of FES, which would be roughly proportional to total sessions dose and total runs dose parameters, may directly cause local changes in muscle mass that increase strength or endurance while reducing atrophy as has been documented in previous studies of FES (Gordon and Mao, 1994; Valli et al., 2002; Gargiulo et al., 2011). Alternately, these changes in SIS Strength may be associated with changes in functional or structural brain characteristics that are not well-captured by changes in motor network LI. If this is the case, further study of brain and behavioral changes with BCI therapy using alternate approaches such as transcranial magnetic stimulation or complementary neuroimaging metrics such as those derived from diffusion tensor imaging or functional connectivity analyses may reveal relationships between brain changes and changes in SIS Strength. In either case, brain-based neuroimaging measures such as LI may be a better indicator of behaviors that are improved in association with neuromodulatory changes, while administered therapy dose may be more predictive of behavioral gains in other domains where improvement cannot be related to brain-based measures but is instead shown to respond directly to differences in BCI therapy administration parameters.

With BCI therapies being developed as an option for stroke patients who have reached a functional plateau with traditional rehabilitation (Broetz et al., 2010; Prasad et al., 2010; Caria et al., 2011; Shindo et al., 2011; Liu et al., 2012; Takahashi et al., 2012; Ang et al., 2014a,b; Mukaino et al., 2014; Ono et al., 2014), the persistence of both brain-associated and brain-independent pathways to functional gains into the chronic stage of stroke recovery is critical. The meta-analysis by Lohse et al. (2014) examining potential effects of stroke chronicity on the dose-response relationships identified showed that the pattern of increased improvement following increased therapy was not affected by stroke chronicity. This finding further supports the idea that additional neuroplastic recovery potential remains even in chronic stroke when traditional therapies have left stroke patients at a functional plateau (Cramer, 2010; Lohse et al., 2014). Although the data in this preliminary set of analyses is insufficient to draw conclusions regarding the overall efficacy of the BCI therapy approach used, group-level improvements meeting MCID estimates were observed at mid-therapy or post-therapy for each of the SIS domains examined. To observe such improvements in this cohort, composed mostly of subjects in the chronic stage of stroke recovery, also supports the hypothesis that additional recovery is possible through the use of such newer rehabilitative approaches even after traditional therapies have stopped yielding significant gains.

The findings relating therapy dose to brain and behavior changes observed in this study suggest that therapy dose and intensity may meaningfully affect the degree of change facilitated by BCI therapy while the frequency with which these therapy sessions are administered may be less likely to affect the overall brain and behavioral changes achieved. These distinctions may be important to consider when designing future studies of BCI therapy and when establishing guidelines for the clinical implementation of therapy with such devices. One component of bringing newer therapies from experimental to clinical settings will be a better understanding of the dose-response relationships for newer classes of therapies. This knowledge is needed to inform the design of future efficacy studies as well as the establishment of clinical guidelines for the use of these developing therapy modalities.

While this study provides a preliminary look at dose-response relationships that may be used to guide future work, there are some limitations that should be acknowledged. These include the heterogeneity of the subjects studied (**Table 2**) and the relatively limited sample size (*n* = 16), which limited our ability to conduct meaningful subanalyses to identify differences in dose-response relationships among subgroups of stroke patients. As has been observed in previous stroke rehabilitation dose-response studies, there may be significant dose-response relationships that take effect only within specific subpopulations of stroke survivors not evident when analyzing the cohort as a whole (Lincoln et al., 1999; Parry et al., 1999). Nevertheless, the presence of the effects observed across this heterogeneous cohort may guide the design of future studies using BCI technology where heterogeneous groups are recruited. This may also allow for a beneficial broader generalization of findings when guiding therapy recommendations for future clinical stroke patients seeking BCI-based therapies. Future work will benefit from the study of larger and more homogenous cohorts so that differential dose-response relationships among subpopulations can be identified.

Although therapy parameters varied from subject to subject, the relatively limited range in which the parameters examined occurred within the cohort may also have precluded the ability of these analyses to detect a true effect. For example, while this study identified no differences between groups receiving low vs. high BCI therapy frequency, there were no individuals studied who received four or more therapy sessions per week. This limits the degree to which these findings may be extrapolated beyond the ranges present. Further investigation into parameters beyond these ranges is needed because similar BCI training has been shown to produce functional benefit in other studies with frequencies as high as five times per week (Ramos-Murguialday et al., 2013; Mukaino et al., 2014; Ono et al., 2014).

One other limitation is the retrospective nature of the data analysis. In these analyses, we have attempted to investigate the influence of individual differences in de facto BCI therapy parameters on outcomes among stroke patients adhering to the same general group treatment guidelines. This approach follows from previous works that have examined dose-response relationships revealed by individual differences in therapy completion or intensity after all subjects had been assigned a single more general treatment plan (Dube et al., 2012). In our experience, circumstantial factors were largely independent of subject therapy schedules, but it is possible that these analyses remain unable to account for unknown confounding factors. The de facto dosing of BCI therapy in this study resulted from a combination of random events and individual needs. Future prospective studies using more rigidly defined dosing parameters will be needed to better characterize and understand BCI therapy dose-response relationships. However, it is important to remember that such parameters will inevitably need to accommodate individual patient needs when attempting to implement similar therapies using BCI devices in real-world clinical practice.

## Author Contributions

BY assisted in subject recruitment, data collection, data analysis, and writing. ZN assisted with data collection and data analysis. LW assisted with data collection and data analysis. AR assisted with data collection. JS assisted with subject recruitment and data collection. VN assisted with subject recruitment, data collection, data analysis, and writing. MT provided TDU hardware and expertise. DE assisted with study design and data analysis. KC assisted with subject recruitment. JS assisted with study design, subject recruitment, and manuscript editing. JW is one of two lead PI’s on this project and supervised the technical and engineering aspects of the work. VP is one of two lead PI’s on this project and supervised the neuroimaging and neuroscience aspects of this work.

## Conflict of Interest Statement

There is one patent pending on the closed-loop neurofeedback device used for the therapy administered in this study (Pending US Patent Application No. 12/715,090). This patent was filed jointly by the two lead investigators Justin C. Williams and Vivek Prabhakaran. Otherwise, the authors have no conflicts of interest to report, as this research was conducted in the absence of commercial and financial relationships that might compromise the integrity of the results reported herein.
