# Supplementary material for: Dose-response relationships using brain–computer interface technology impact stroke rehabilitation
Source: Front Hum Neurosci. 2015 Jun 23;9:361. doi: 10.3389/fnhum.2015.00361 (PMC4477141; doi:10.3389/fnhum.2015.00361)
Supplement: Supplementary file 1 [file Data_Sheet_1.DOCX]

**Supplementary Methods**

*Laterality Index (LI) calculation parameters*

Because LI values can be threshold dependent (Pillai and Zaca, 2011) and may vary as a function of which regions are included in the masks used for calculation, LI was calculated using two additional sets of ROI masks – one covering the whole brain and another covering only the motor cortex. Each mask set was then evaluated at one relatively permissive (t=2.003, p<0.05) threshold and one relatively stringent threshold (t=4, p<0.0001). The motor network mask used for analyses presented in the main manuscript was also evaluated at the lower permissive threshold with those additional results presented here. This approach of evaluating multiple mask and threshold sets is consistent with previously described methods (Young et al., 2014c) and allows for the identification of effects that persist across mask-threshold combinations. Such effects that are found to be relatively consistent across multiple mask and threshold combinations may therefore be more reliable markers for tracking neuroplastic change.

*LI Mask Set Components*

Each mask in the Motor Network Mask Set used for analyses presented in the main manuscript included brain areas canonically associated with movement of the contralateral hand. Thus, for this set of masks the left motor network mask comprised areas of the left sensorimotor cortex, the left thalamus, and the right cerebellum while the right motor network mask comprised areas of the right sensorimotor cortex, the right thalamus, and the left cerebellum.

Each mask in the Motor Cortex Mask Set used for analyses presented in the Supplementary Results was a subset of the Motor Network Mask containing only the cortical areas contained in the corresponding side of the Motor Network Mask. Thus, for this set of masks the left motor cortex mask comprised areas of the left sensorimotor cortex while the right motor cortex mask comprised areas of the right sensorimotor cortex.

Each mask in the Whole Brain Mask Set used for analyses presented in the Supplementary Results encompassed all ipsilateral cortical and subcortical structures except for the cerebellum along with cerebellar structures from the contralateral side of the brain. Thus, for this set of masks the left whole brain mask comprised the left cortex, left subcortical structures, and the right cerebellum while the right whole brain mask comprised the right cortex, right subcortical structures, and the left cerebellum.

*Statistical analyses*

A generalized estimating equations (GEE) (Ballinger, 2004) approach was used to examine correlations between changes in LI from baseline pre-therapy values with therapy sessions dose, therapy runs dose, and therapy intensity for each of these additional mask-threshold combinations. A GEE approach was also used to investigate brain-behavior relationships, examining correlations between changes in LI values calculated using each of these additional mask-threshold combinations with changes in behavioral measures. As these analyses were not part of the main hypothesis investigated in the main text, results are presented in this supplement uncorrected for multiple comparisons.

**Supplementary Results**

*Neuroimaging outcome measures*

Group LI values obtained from each additional mask-threshold combination at each time point are presented in Table S1.

Table S1. Supplementary Group neuroimaging outcome measures. Thr = threshold. Numbers shown in X (Y) format represent Mean (Standard Deviation).

| Mask Set | Thr | Baseline | Mid-Therapy | Post-Therapy | One Month |
| --- | --- | --- | --- | --- | --- |
| Whole Brain | 2 | -0.059 (0.310) | 0.097 (0.258) | -0.216 (0.346) | -0.058 (0.118) |
| Whole Brain | 4 | 0.058 (0.478) | 0.189 (0.390) | -0.212 (0.495) | -0.080 (0.151) |
| Motor Network | 2 | 0.086 (0.478) | 0.171 (0.460) | -0.110 (0.482) | 0.135 (0.436) |
| Motor Cortex | 2 | 0.041 (0.590) | 0.233 (0.598) | -0.114 (0.511) | 0.062 (0.491) |
| Motor Cortex | 4 | 0.253 (0.738) | 0.215 (0.719) | -0.108 (0.586) | 0.156 (0.634) |

*BCI therapy dosing parameter correlations with brain changes (fMRI-LI)*

GEE analyses examining potential relationships between changes in LI values and BCI therapy parameters are summarized in Table S2.

Table S2. Correlations between BCI Therapy Doses and Changes in Neuroimaging Measures. LI = Laterality Index, Thr = threshold, Est = Estimated. Figures in bold denote relationships achieving an uncorrected p<0.001.

| LI Calculation Parameters | | Therapy Sessions Dose | | Therapy Runs Dose | | Therapy Intensity | |
| --- | --- | --- | --- | --- | --- | --- | --- |
| Mask Set | **Thr** | **Est β** | **p-value** | **Est β** | **p-value** | **Est β** | **p-value** |
| Whole Brain | 2 | -0.0566 | **p<0.001** | -0.0019 | **p<0.001** | 0.0024 | 0.6192 |
| Whole Brain | 4 | -0.0385 | **p<0.001** | -0.0013 | 0.0012 | -0.0043 | 0.3040 |
| Motor Network | 2 | -0.0638 | 0.0023 | -0.0020 | 0.0094 | 0.0018 | 0.7799 |
| Motor Cortex | 2 | -0.0569 | 0.0019 | -0.0017 | 0.0048 | 0.0061 | 0.3603 |
| Motor Cortex | 4 | -0.0371 | 0.0317 | -0.0013 | 0.0168 | -0.0136 | 0.1220 |

*Brain change (fMRI-LI) correlations with changes in behavioral measures*

GEE analyses examining potential relationships between changes in LI values and changes in the behavioral outcomes studied are summarized in Table S3.

Table S3. Correlations Between Changes in Neuroimaging Measures and Changes in Behavioral Scores. LI = Laterality Index, Thr = threshold, SIS = Stroke Impact Scale, ADL = Activities of Daily Living, ARAT = Action Research Arm Test, 9-HPT = 9-Hole Peg Test. Figures in bold denote relationships achieving an uncorrected p<0.001.

| Behavioral Measure | LI Calculation Parameters | | Estimated β | p-value |
| --- | --- | --- | --- | --- |
|  | **Mask Set** | **Thr** |  |  |
| SIS Strength | Whole Brain | 2 | 3.8815 | 0.6174 |
|  | Whole Brain | 4 | 36.2709 | 0.5552 |
|  | Motor Network | 2 | 7.1555 | 0.1632 |
|  | Motor Cortex | 2 | 19.3578 | 0.1952 |
|  | Motor Cortex | 4 | 2.6903 | 0.2935 |
| SIS ADL | Whole Brain | 2 | -2.8559 | 0.4012 |
|  | Whole Brain | 4 | -4.6137 | 0.1315 |
|  | Motor Network | 2 | -4.3123 | 0.0139 |
|  | Motor Cortex | 2 | -4.0391 | 0.0061 |
|  | Motor Cortex | 4 | -5.5511 | 0.2413 |
| SIS Hand Function | Whole Brain | 2 | 17.4671 | 0.2741 |
|  | Whole Brain | 4 | -0.3948 | 0.9434 |
|  | Motor Network | 2 | 24.7184 | **p<0.001** |
|  | Motor Cortex | 2 | 11.7744 | 0.3265 |
|  | Motor Cortex | 4 | -25.3752 | 0.1634 |
| ARAT | Whole Brain | 2 | 6.9124 | 0.1049 |
|  | Whole Brain | 4 | 3.4371 | 0.0072 |
|  | Motor Network | 2 | 2.2870 | 0.1374 |
|  | Motor Cortex | 2 | 1.8921 | 0.1685 |
|  | Motor Cortex | 4 | 1.7036 | 0.2206 |
| 9-HPT | Whole Brain | 2 | 3.9802 | 0.8282 |
|  | Whole Brain | 4 | -2.3837 | 0.9271 |
|  | Motor Network | 2 | -4.9007 | 0.6297 |
|  | Motor Cortex | 2 | -4.9996 | 0.6859 |
|  | Motor Cortex | 4 | 17.5464 | 0.0784 |
